# Efficacy of lanreotide Autogel® administered every 4–8 weeks in patients with acromegaly previously responsive to lanreotide microparticles 30 mg: a phase III trial

**DOI:** 10.1111/j.1365-2265.2006.02595.x

**Published:** 2006-09

**Authors:** T Lucas, R Astorga, and the Spanish–Portuguese Multicentre Autogel Study Group on Acromegaly

**Affiliations:** * Service of Endocrinology, Puerta de Hierro Madrid, Spain; † Service of Endocrinology, Hospital ‘Virgen del Clínica Rocío’ Sevilla, Spain

## Abstract

**Objective and design:**

Depot somatostatin analogues are well accepted as either adjuvant or primary therapy for acromegaly, and their long dosage intervals facilitate adherence to treatment. Our objective was to evaluate whether lanreotide Autogel® 120 mg, every 4–8 weeks, was as effective in controlling acromegaly as lanreotide microparticles 30 mg, every 1–2 weeks.

**Patients design and measurements:**

Patients who had used lanreotide microparticles 30 mg, ≥ 2 months prestudy, and had responded to treatment were recruited to this open, prospective, multicentre phase III trial. Three to five injections of lanreotide Autogel® 120 mg were administered. Lanreotide Autogel® 120 mg was injected every 4, 6 or 8 weeks in patients previously receiving lanreotide microparticles every 7, 10 or 14 days, respectively. GH and insulin-like growth factor (IGF)-1 levels were assessed one dosing interval after the final injections.

**Results:**

Ninety-eight patients were enrolled and 93 completed. Steady-state GH concentrations demonstrated similar efficacy between the formulations (upper 95% confidence interval of the quotient, 77·7%). Mean (SE) GH levels were lower with lanreotide Autogel® than with lanreotide microparticles (3·8 (0·5) *vs* 4·3 (0·5) ng/ml; *P* < 0·001). GH levels < 2·5 ng/ml were observed in 54% and 46% of patients; 40% and 35% having GH < 2·5 ng/ml and normalized IGF-1 with lanreotide Autogel® and microparticles, respectively. Symptoms were controlled better with lanreotide Autogel® and treatment was well accepted.

**Conclusions:**

Lanreotide Autogel® 120 mg every 4–8 weeks, is at least as effective and as well tolerated in acromegaly as lanreotide microparticles 30 mg injected every 7–14 days.

## Introduction

The efficacy of somatostatin analogues in the treatment of acromegaly is determined by the number, distribution and activity of somatostatin receptors in the pituitary GH-producing adenoma.^1^,^2^ Somatostatin analogues are indicated principally for the treatment of acromegaly that remains active after transsphenoidal surgery, whether or not the patient has also undergone radiotherapy. Somatostatin analogues have, however, also been used in selected patients as primary treatment, for example in those whose vision is not compromised by the presence of the tumour or whose tumours are unlikely to be excised completely (e.g. macroadenomas, invasive adenomas).^3^,^4^ These analogues can also be beneficial prior to surgery, and tumour size is reduced in 30% of patients given lanreotide for 1–3 months before transsphenoidal surgery. 5

Long-term use of somatostatin analogues has been made easier by the development of prolonged-release formulations.^6^ Octreotide is available as a 4-week formulation (octreotide LAR)^7^ and lanreotide as either a 1–2 week microparticle (MP) formulation or a 4-week aqueous Autogel® (ATG) formulation.^8^–^11^ When given by deep subcutaneous (sc) injection every 4 weeks at 60, 90 or 120 mg, the Lan ATG formulation is as effective at controlling levels of GH and IGF-1 as either the Lan MP 30 mg formulation^8^,^12^ or octreotide LAR.^13^–^15^

By maintaining the same total monthly dose, but with fewer injections, the prolonged-release formulations offer clear treatment advantages. The aim of our study (study A92 52030 046) was to evaluate whether the dosing interval of Lan ATG could be extended beyond 4 weeks, without compromising efficacy or safety. Thus, we investigated whether Lan ATG 120 mg administered at intervals of 4, 6 or 8 weeks, had no less efficacy than Lan MP 30 mg administered every 7–14 days. We also compared the safety of the two formulations.

## Patients and methods

### Patients

Patients who were eligible for this study had a diagnosis of acromegaly, defined as GH levels over 2 ng/ml after an oral glucose tolerance test, and IGF-1 concentrations elevated above the age- and sex-matched normal range. Patients whose last radiotherapy was over 10 years previously were required to have had active acromegaly confirmed in the 12 months before inclusion in the study. In cases of incomplete tumour excision, as evidenced by imaging, one blood sample showing GH levels over 5 ng/ml qualified as acromegaly. Eligible patients had responded to somatostatin analogues, defined as a reduction in GH of 50% or more from baseline, normalization of IGF-1 or a reduction of 30% or more, after a minimum period of 2 months of treatment. All patients had used Lan MP 30 mg at fixed intervals of administration, for a minimum of 2 months immediately before their inclusion. Patients were excluded if they had received treatment with octreotide LAR in the last 5 months; received treatment with dopaminergic agonists in the 2 months before the study; or underwent radiotherapy for acromegaly within 2 years prior to starting the study. All patients gave written informed consent, and the study was approved by the Ethics Committee of each participating centre and by the Health Authorities of the participating countries. The study was carried out in accordance with the guidelines of the Declaration of Helsinki.

### Study design

This study was a phase III, open, multicentre, prospective study, in which patients served as their own controls as they switched between treatments. Patients were injected with Lan ATG 120 mg at a frequency determined by their dosage interval with Lan MP 30 mg at the time of their inclusion. This interval had been chosen by the investigators in order to optimally control their disease. The total monthly dose of Lan was, therefore, kept constant as patients switched between formulations. Lan ATG 120 mg was injected every 8 weeks when the dosing interval with Lan MP 30 mg was every 14 days, and every 6 or 4 weeks for Lan MP administered every 10 and 7 days, respectively. There was no washout period. Three injections of Lan ATG were planned for each treatment group, but some patients, whose vacation period interfered with the trial completion, received five injections. There was no dose titration. The duration of treatment with Lan ATG ranged from 84 to 210 days. Lan MP 30 mg was given by intramuscular injection, whereas Lan ATG 120 mg was given by deep sc injection.

### Efficacy and safety assessments

Blood samples were taken immediately before the final injection of Lan MP, before the final injection of Lan ATG and at times after the final injection of Lan ATG, according to the dosing interval. For patients with 8-week dosing, samples were taken at weeks 1, 2, 5 and 8 after the final Lan ATG injection; for 6-week dosing, samples were taken at weeks 1, 2, 4 and 6, and for 4-week dosing, samples were taken 1, 2, 3 and 4 weeks after the last Lan ATG injection. At each sampling time, blood was taken every 30 min over a 4-h period. All samples were used to determine GH concentrations, whereas only the first samples from each series were used for IGF-1 and Lan assessment.

Symptoms of acromegaly were assessed at the beginning and at the end of the study. Symptoms such as headache, hyperhidrosis, asthenia and arthralgia were graded as: none, mild (present but does not interfere with normal daily activity), moderate (normal daily activity difficult) or severe (impedes normal daily activity). Patients also expressed their satisfaction with the treatment at the end of the study using a visual analogue scale ranging from 1 to 10, with the score of 10 as maximum acceptance of treatment.

Both systemic and local tolerability were recorded after each administration. Systemic tolerability included the incidence and severity of nausea, diarrhoea, abdominal distension, abdominal pain and cramps. Tolerability at the injection site was monitored 30 min after each injection at which investigators noted the presence of heat, erythema or local indurations. Patients evaluated any pain relating to the administration of the drug using a visual analogue scale ranging from 0, absence of pain to 10, maximum pain imaginable. All adverse events reported were recorded. Biliary ultrasounds were performed and standard haematological and biochemical tests were undertaken at the beginning and at the end of the study.

### Analytical determinations

All analytical assays were processed centrally. GH concentrations were measured with an immunoradiometric assay from Nichols Institute Diagnostics (San Juan de Capistrano, CA), with a detection limit of 0·02 ng/ml and intra- and interanalysis variation coefficients of 5·1% and 14·9%, respectively. IGF-1 was measured with an immunoradiometric assay, also from Nichols Institute Diagnostics, with a detection limit of 4·6 ng/ml and intra- and interanalysis variation coefficients of 5·9% and 5·9%, respectively. Serum levels of Lan were determined using an immunoradiometric method validated previously, with a detection limit of 0·078 ng/ml and coefficients of variation between 2·3% and 13·6%.^16^

### Statistical evaluation

We tested the hypothesis that the new prolonged-release formulation Lan ATG 120 mg administered every 4, 6 or 8 weeks is no less effective than Lan MP 30 mg administered every 7–14 days in the control of acromegaly. To determine similar efficacy, steady-state GH concentrations with Lan ATG and Lan MP were determined, and the quotient of the GH concentrations calculated. Noninferiority would be demonstrated if the 95% confidence interval (CI) of this ratio had an upper limit below 125%, assuming log transformation of the data. Differences between matched values were evaluated using the paired *t*-test or the Wilcoxon-signed rank test, depending on the necessity of a parametric test. The McNemar test was used for dichotomy variables.

Using the criteria established for bioequivalence studies,^17^ a sensitivity of 20% and a coefficient of variation of 80·48%, we calculated that a sample of 102 patients would be necessary to guarantee a study power of 80%. Only 98 patients were enrolled, however, as a preliminary analysis with 74 patients showed that statistical significance had already been obtained. Both the per-protocol (PP) population and those patients who had completed the study were included in the analyses.

## Results

### Patients

A total of 98 patients with acromegaly were enrolled in centres across Spain and Portugal. One patient withdrew consent before receiving any medication; the remaining 97 patients who received at least one injection of Lan ATG 120 mg constituted the safety population. Demographic characteristics for the safety population are shown in Table 1. Three of the safety population did not complete the study and one patient had no valid hormonal determinations after treatment; thus, there were 93 patients in the completers group. Eighty patients completed the study without protocol deviations and formed the PP population.

**Table 1:** Demographic and baseline characteristics of patients (safety population, *n* = 97)

Male/female, *n*	44/53
Age, years	50·6 (13·6)
Body mass index, kg/m^2^	30·1 (5·1)
Cholelithiasis, *n* (%)	25/90 (27·8)
High blood pressure, *n* (%)	36/97 (37·1)
Diabetes mellitus, *n* (%)	23/97 (23·7)
Systolic blood pressure, mmHg	134·1 (19·6)
Diastolic blood pressure, mmHg	82·7 (12·2)

Data are presented as mean (SD) unless stated otherwise.

All patients comprising the safety population had been diagnosed with acromegaly a mean (± SE) of 9·4 ± 0·7 years before inclusion in the study. Previous treatment for acromegaly included adenomectomy in 76/97 (78%) patients, whereas 53/97 (55%) had received pituitary radiotherapy for a minimum of 2 years before inclusion. The mean (± SE) duration of somatostatin analogue treatment prior to study inclusion was 3·1 ± 0·3 years. Lan ATG 120 mg was injected every 4, 6 or 8 weeks for 13 (13%), 31 (32%) and 53 (55%) patients, respectively.

### Hormonal control and drug levels

**Fig. 1:**
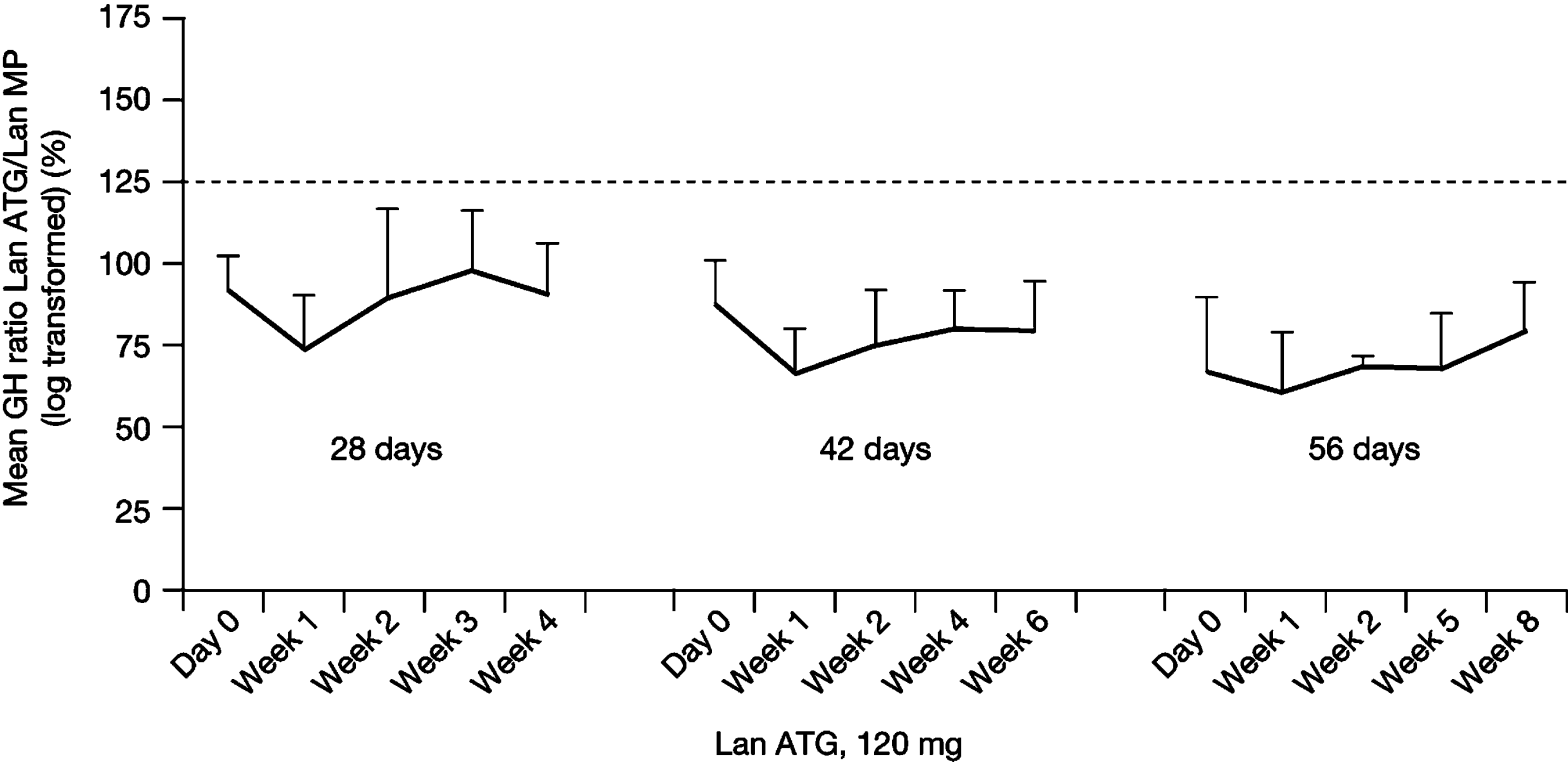
Mean ratio of GH levels and upper limit of confidence interval (95%) for the mean GH ratio at different times (weeks after the last Lan ATG injection) throughout the last dosage interval (log-transformed values).

Similar efficacy between the different formulations was shown by the upper limit of the 95% CI for the mean ratio of GH concentrations, with Lan ATG 120 mg over Lan MP 30 mg being less than 125%. This was true for both the completers (upper 95% CI, 77·7%) and the PP population (upper 95% CI, 76·8%). Also, similar efficacy between Lan ATG 120 mg and Lan MP 30 mg was shown separately for each of the 4-, 6- and 8-week dosing groups for each time-point analysed (Fig. 1). A check for homogeneity between three and five injections was performed.

Mean GH concentrations after treatment with Lan MP 30 mg, and at times after the final dose of Lan ATG 120 mg are shown in Table 2. For both the PP population (*P <* 0·001) and the completers group (*P <* 0·001), mean GH levels were lower one dosing interval after the final injection of Lan ATG 120 mg than one dosing interval after treatment with Lan MP 30 mg. Control of GH levels below 2·5 ng/ml was achieved in 46% of patients with Lan MP 30 mg and in 54% of patients with Lan ATG 120 mg (*P =* 0·052). Whereas 35% of patients had both GH levels below 2·5 ng/ml and IGF-1 normalized in accordance with age and sex with Lan MP 30 mg this level of control was present in 40% of patients after treatment with Lan ATG 120 mg. Plasma concentrations of IGF-1 did not change significantly over the course of the study. During treatment with Lan MP 30 mg, the mean plasma concentration of IGF-1 was 423 ± 30 ng/ml in men and 336 ± 24 ng/ml in women, compared with 414 ± 32 in men and 314 ± 23 ng/ml in women after treatment with Lan ATG 120 mg. The percentage of patients with IGF-1 within the normal range for age and sex remained unchanged between baseline (55%) and at the end of the study (56%).

**Table 2:** Concentrations of GH after the last dose of Lan MP, 30 mg and at times after the last dose of Lan ATG, 120 mg

	GH (ng/ml)	
	
	Per protocol population (*n* = 80)	Completers (*n* = 93)
Lan MP, 30 mg		
One dosing interval after final injection	4·1 (0·5)	4·3 (± 0·5)
Lan ATG, 120 mg		
Immediately before final injection	3·6 (± 0·6)***	3·8 (± 0·5)***
+1 week after final injection	2·5 (± 0·3)***	2·5 (± 0·3)***
+2 weeks after final injection	2·2 (± 0·3)***	2·5 (± 0·4)***
+3–5 weeks† after final injection	2·9 (± 0·4)***	3·0 (± 0·4)***
+1 dosing interval‡ after final injection (end of study)	3·7 (± 0·5)**	3·8 (± 0·5)***

Data are presented as mean (± SEM).

** P < 0·001;

*** P≤ 0·0001 vs baseline visit.

† 3, 4 or 5 weeks from last dose for 4-, 6- or 8-week dosing, respectively.

‡ 4, 6 or 8 weeks from the last dose for 4-, 6- or 8-week dosing.

Table 3 summarizes the plasma concentrations of Lan according to dosage intervals. There was no difference in minimum concentrations of Lan between the Lan MP and the Lan ATG formulations when dosing was equivalent. Similarly, Lan concentrations did not differ at 4 and 6 weeks, irrespective of whether three or five injections had been administered (Table 3). All patients on 8-week dosing had received three doses.

**Table 3:** Minimum serum concentration of Lan by dosing interval of Lan MP and Lan ATG, and by number of doses of Lan ATG (completers, *n* = 93)

	Minimum serum Lan (ng/ml)	
	
Dosing interval	Lan MP, 30 mg	Lan ATG, 120 mg
Lan MP 7 days/Lan ATG 4 weeks		
Three doses of Lan ATG (*n* = 8)	2·6 (0·4)	2·4 (0·3)
Five doses of Lan ATG (*n* = *5*)	2·1 (0·4)	2·4 (0·3)
Lan MP 10 days/Lan ATG 6 weeks		
Three doses of Lan ATG (*n* = *11*)	1·8 (0·2)	1·9 (0·2)
Five doses of Lan ATG (*n* = *16*)	2·0 (0·2)	2·2 (0·2)
Lan MP 14 days/Lan ATG 8 weeks		
Three doses of Lan ATG (*n* = 53)	1·4 (0·1)	1·6 (0·1)

Data are presented as mean (SEM). Samples were taken one dosing interval after the last dose of study.

### Symptom control

**Fig. 2:**
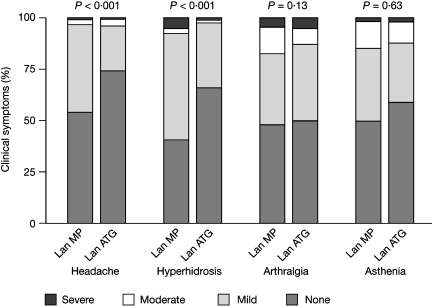
Presence and intensity of the symptoms of acromegaly after treatment with lanreotide microparticles (Lan MP), 30 mg, or Lan ATG, 120 mg. The percentage of patients reporting symptoms at the selection visit was compared with the percentage of patients having symptoms at the final visit through a McNemar's test.

The symptoms of acromegaly were generally of mild or moderate intensity. Overall, greater symptom control was noted at the end of treatment with Lan ATG 120 mg than with Lan MP 30 mg (Fig. 2). The improvement was statistically significant with regard to hyperhidrosis (*P <* 0·001) and headache (*P <* 0·001).

### Acceptance of treatment

Lan ATG 120 mg was well accepted by patients, with a mean acceptance score of 8·3 (95% CI, 7·9–8·7), from a maximum possible score of 10. Results were similar between dosing groups, with a mean (95% CI) of 8·2 (7·0–9·3), 8·4 (7·5–9·2) and 8·3 (7·9–8·8), for administration every 4, 6 and 8 weeks, respectively.

### Adverse events

There were no serious adverse events related to either Lan ATG 120 mg or Lan MP 30 mg. One patient died while participating in the study, but this was not considered related to the study drug. The patient had been diagnosed with acromegaly 9 years before inclusion in the study and had been receiving regular treatment with Lan MP for 2 years and 4 months without any tolerability problems. He did, however, have a history of dilated cardiopathy and auricular fibrillation. The patient was admitted for coughing and dyspnoea and, while in the hospital, suffered general deterioration and various cardiovascular complications. He died from cardiovascular shock and intravascular coagulation. One patient withdrew because of the persistency of acromegaly symptoms and one patient withdrew because of pain radiating from the left leg after the first dose of Lan ATG.

The most common adverse events were gastrointestinal in nature; 51% of patients reported diarrhoea, 42% flatulence, 41% abdominal pain and 14% nausea at some time during their treatment with Lan ATG. Systemic and local tolerability for the last doses of Lan MP and Lan ATG are shown in Table 4. Gallbladder ultrasound was performed in 90 patients at the beginning of the study and in 84 patients at the last visit. Overall, the prevalence of lithiasis or sludge did not alter over the study (34/90 at selection visit and 32/84 at last visit); whereas six patients developed lithiasis or sludge during the study, these abnormalities were present initially and disappeared during the study period in seven patients. No significant alterations were detected in any of the clinical or analytical parameters evaluated.

**Table 4:** Percentage of patients with digestive adverse events or adverse events at the injection site: comparison after final injections of Lan MP, 30 mg, or Lan ATG (safety population, *n* = 97)

	Lan MP, 30 mg	Lan ATG, 120 mg
Digestive tolerability, %		
Abdominal distension	36	29
Diarrhoea	34	27
Abdominal pain	24	20
Abdominal cramps	13	5
Nausea	11	8
Local tolerability, %		
Induration	19	17
Erythema	4	0
Heat	9	6
Pain	31	18

## Discussion

This study compares the efficacy between Lan ATG 120 mg administered at intervals of 4, 6 or 8 weeks and Lan MP 30 mg administered every 7–14 days in patients with acromegaly who were responsive to treatment with somatostatin analogues.

The longer dosing period with Lan ATG was at least as efficacious in controlling levels of GH as 7–14-day dosing with Lan MP. Similar efficacy was demonstrated by the upper limit of the 95% CI, for the quotient of GH concentrations under Lan ATG and Lan MP falling below 125%. This was also shown for each of the different dosing intervals with Lan ATG and Lan MP, and at each time-point assessed. Furthermore, a trend towards superior control of GH was observed with lan ATG, as levels of GH were significantly lower than in those treated with Lan MP and control of GH, defined as plasma concentrations below 2·5 ng/ml, was better with Lan ATG. Whereas only 46% of patients had GH control with Lan MP this increased to 54% in those treated with Lan ATG, although this just missed statistical significance. The therapeutic objective of GH control below 2·5 ng/ml and concentrations of IGF-1 within the normal range is widely used, as patients with acromegaly who have this level of control have a mortality rate similar to that of the general population.^18^–^23^ This also showed a trend to superiority on lan ATG, with 40% patients showing good biochemical control, rising from 35% on Lan MP.

The proportion of patients with GH and IGF-1 control in the present study is similar to results from previous studies with Lan^8^,^24^,^25^ and octreotide.^26^ Notably, control was achieved in this study even though over half of the patients had an injection frequency of every 8 weeks. These comparisons indicate that patients who are well controlled on Lan ATG can use an extended dosing interval without compromising efficacy. The longer dosing interval is also likely to provide cost savings through fewer injections needing to be given.

As seen in previous studies with Lan and octreotide, a proportion of all patients were poorly responsive, despite receiving high doses of somatostatin analogue. Thus, patients receiving Lan MP every 7 days/Lan ATG every 4 weeks had higher mean GH concentrations than patients with less frequent injections. Poorly responsive patients are likely to have a GH-secreting adenoma with a somatostatin receptor profile that is not preferential to Lan or octreotide, which bind to type 2 and 5 receptors.^27^,^28^ These patients could be candidates for supplementing somatostatin analogue treatment with weekly doses of the GH antagonist, pegvisomant or with a dopaminergic agonist. Both approaches can significantly improve biochemical control of acromegaly.^29^,^30^

Patients recruited to this study had used previous medical, surgical and radiological therapies to control their acromegaly. Among the study population, 78% had undergone adenomectomy and 55% radiotherapy. To minimize any bias from the effects of radiotherapy, patients were excluded if they had completed their treatment in the preceding 2 years. Thus, this exclusion avoided the period in which radiotherapy exerts its maximum effect. For patients who had completed radiotherapy in the previous 10 years, the activity of the disease was documented during the year prior to their inclusion in the study.

The symptoms of acromegaly were less severe during treatment with Lan ATG 120 mg than with Lan MP 30 mg with a significant improvement in headache and hyperhidrosis. Patients showed a high level of acceptance of treatment with Lan ATG 120 mg with a mean score of 8·3 (95% CI, 7·5–9·2) out of a maximum of 10. Acceptance of Lan MP was not assessed.

Side effects were mostly gastrointestinal in nature and of mild to moderate intensity, as reported in previous studies with somatostatin analogues.^8^,^13^,^25^,^31^ Lan ATG showed a trend towards an improved digestive tolerability compared with Lan MP. This has been reported previously and may relate to the pharmacokinetic characteristics of Lan ATG; whereas Lan has a smooth release profile from ATG with a minimal initial burst effect, it is released rapidly from the surface of the MP copolymers, resulting in a peak in the plasma concentration of drug.^8^,^9^,^32^ A second factor that may contribute to improved tolerance with Lan ATG is that in the clinical experience of the study investigators, the incidence of gastrointestinal symptoms is usually limited to the first days following administration of a somatostatin analogue. Extension of the dosage interval, with a concomitant reduction in the number of administrations, may therefore provide an overall reduction in the number of digestive events.

In conclusion, treatment with Lan ATG administered every 4–8 weeks was at least as effective and well tolerated as Lan MP administered every 7–14 days in patients with active acromegaly. There was a good level of acceptance of Lan ATG. The longer dosing interval of Lan ATG 120 mg therefore maintains the same overall monthly dose as Lan MP 30 mg but with four times fewer injections, and thus has a benefit in terms of cost and patient compliance.
